# Murine Extraparenchymal Neurocysticercosis: Appropriate Model for Evaluating Anthelminthic and Anti-Inflammatory Treatment Schedules

**DOI:** 10.3390/tropicalmed9090215

**Published:** 2024-09-16

**Authors:** Vinícius Tadeu Oliveira, Tatiane de Camargo Martins, Renato Tavares Conceição, Diego Generoso, Vânia Maria de Vasconcelos Machado, Sabrina Setembre Batah, Alexandre Todorovic Fabro, Marco Antônio Zanini, Edda Sciutto, Agnès Fleury, Pedro Tadao Hamamoto Filho

**Affiliations:** 1 Department of Neurosciences and Mental Health, Botucatu Medical School, UNESP—São Paulo State University, Botucatu 18618-686, Brazil; 2 Department of Veterinary Surgery and Animal Reproduction, School of Veterinary Medicine and Animal Science, UNESP—São Paulo State University, Botucatu 18618-686, Brazil; 3 Department of Pathology and Legal Medicine, Ribeirão Preto Medical School, USP—São Paulo University, Ribeirão Preto 14049-900, Brazil; 4 Department of Immunology, Institute of Biomedical Investigations, UNAM—Universidad Nacional Autónoma de Mexico, Ciudad de México 14269, Mexico; 5 Department of Medical Genomics and Environmental Toxicology, Institute of Biomedical Investigations, UNAM—Universidad Nacional Autónoma de Mexico, Ciudad de México 14269, Mexico; 6 INNN—National Institute of Neurology and Neurosurgery, Ciudad de México 14269, Mexico

**Keywords:** neurocysticercosis, *Taenia crassiceps*, albendazole, dexamethasone, inflammation, experimental model

## Abstract

Background: Experimental models of neurocysticercosis (NCC) are helpful for an improved understanding of the pathophysiological mechanisms of human diseases and for testing novel therapeutic approaches. Controlling inflammation without reducing the effectiveness of anthelmintics is an important challenge in treating neurocysticercosis. This study investigates the effects of currently used drugs (Albendazole and Dexamethasone) in treating murine extraparenchymal NCC. Methods: Twenty-two rats were inoculated with *Taenia crassiceps* in the subarachnoid space. The animals underwent magnetic resonance imaging to ascertain the success of infection 3 months after inoculation. The infected animals were randomly assigned to one of the three groups (five rats each): control (no treatment), Albendazole (ABZ), or Albendazole + Dexamethasone (ABZ + DXM) for 14 days. The animals were subsequently euthanised for morphological assessment 2 weeks after the end of treatment. Results: Macroscopically integrated cysts were found in all animals. The ABZ + DXM animals demonstrated lower ventricular sizes, lymphocyte infiltration rates, and immunopositivity for IL-6, with statistical differences in lymphocytes within the arachnoid region. Conclusions: This experimental model, which has previously shown similarities to human infections, is also helpful in reproducing the morphological changes upon treatment with Albendazole and Dexamethasone.

## 1. Introduction

Neurocysticercosis (NCC) is a common parasitic disease of the central nervous system. Despite recent advancements in sanitary conditions that have decreased the incidence of this disease, it remains endemic to several low- and middle-income countries [1,2]. The larvae can lodge within the brain parenchyma or in the cerebrospinal fluid compartments (brain ventricles and subarachnoid space), causing two different forms of the disease, parenchymal NCC (P-NCC) and extraparenchymal NCC (EP-NCC), respectively. The latter is the most severe form of the disease because it leads to life-threatening symptoms such as increased intracranial pressure and torpid response to cysticidal treatment [3,4,5].

Experimental NCC models are helpful for an improved understanding of the pathophysiological mechanisms of human diseases and for testing new therapeutic approaches [6]. Various NCC experimental models have been developed recently using different hosts, parasites, and inoculation approaches [7,8,9,10,11]. It has been demonstrated that the low invasive murine model of extraparenchymal NCC induced by injecting *Taenia crassiceps* into the subarachnoid space highly resembles the immune–inflammatory features of human disease [11,12,13]. Inflammation mediates parasite destruction but can also be harmful to the patients, contributing to the development of intracranial hypertension and other severe symptoms that can lead to death [14]. Thus, a target for testing new therapeutic approaches involves fine-tuning to control the inflammation promoted by cyst destruction. Prior to testing new therapeutic regimens, it is relevant to deeply understand the pathophysiologic mechanisms involved in the currently used anthelminthic and anti-inflammatory drugs. This study investigated the inflammatory and anti-inflammatory effects of the currently used drugs (Albendazole and Dexamethasone) in treating murine extraparenchymal NCC.

## 2. Materials and Methods

The local Institutional Review Board approved this study and the current legislation on experimental research using animals was followed. Twenty-two Wistar 6-week-old male rats (*Rattus novergicus*) were used. The animals were kept at room temperature (21 °C), with free access to food and water, under 12/12 h of light/dark cycles. The rats were anaesthetised with a 0.1 mL/kg of ketamine (100 mg/mL) and xylazine (20 mg/mL) mixture before treatment. Each animal was injected with 50 *T. crassiceps* cysts in 0.2 mL of saline (0.9%) into the subarachnoid space between the occipital bone and the first cervical vertebra using a 24-gauge needle. Details of the method employed for parasite inoculation have been previously published [11]. The rats were evaluated using magnetic resonance imaging (MRI) 3 months after inoculation to verify the parasite establishment. The criterion for a successful infection was the development of enlarged ventricles (hydrocephalus). The MRI protocol consisted of 0.6 mm slice thickness, using T2 echo-gradient acquisition (echo time, 5 ms; repetition time, 10 ms) on 0.25 T equipment (Esaote, model Vet-MR, Santo André, São Paulo, Brazil).

**Figure 1:**
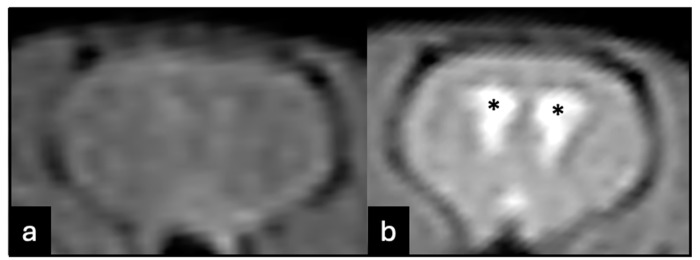
Magnetic resonance imaging of animals with unproven (**a**) or proven (**b**) disease. Animals that did not develop hydrocephalus were excluded. The hydrocephalus was considered as evident ventricle enlargement (*).

Rats with enlarged ventricles on MRI (Figure 1) were randomly assigned to one of the three groups (five rats each): control (no treatment), Albendazole (ABZ), or Albendazole + Dexamethasone (ABZ + DXM). Animals in the ABZ group received 30 mg/kg of Albendazole via gavage daily for 14 days. The animals in the ABZ + DXM group received 30 mg/kg Albendazole and 0.5 mg/kg Dexamethasone via gavage daily for 14 days. Two weeks after terminating the treatment, the rats were euthanised using a ketamine and xylazine overdose. Cardiac perfusion was performed with 0.9% saline, followed by brain fixation in 10% buffered formalin. After the brains were harvested, they were sliced at the level of the optic chiasm. The distance between the frontal horns and the diameter of the brain parenchyma at the same level were measured to assess post-treatment hydrocephalus (ventricular index). The slices were subsequently incubated in formalin for 24 h, dehydrated with increasing alcohol concentrations, cleared in xylene, and embedded in paraffin.

The blocks were cut into 5 μm sections for haematoxylin–eosin staining and into 3 μm sections for immunohistochemical staining for anti-IL-6 within the brain tissue. To quantify the lymphocytes (fractional area with cells) in the basal arachnoid and periventricular regions (striatum and lateral septal nucleus), morphometric analysis was performed. The pro-inflammatory state within the arachnoid and periventricular zones was evaluated by estimating the IL-6 expression levels. The slices were dewaxed in xylene and rehydrated using decreasing alcohol concentrations. Antigen recovery included heating the slides at 98 °C with citrate buffer (pH 6.0) for 30 min and subsequently incubating in 0.3% hydrogen peroxide at room temperature for endogenous tissue peroxidase block. The membranes were subsequently washed with Tris-buffered saline/Tween 20 (TBST) at pH 7.5 and incubated with primary antibodies against IL-6 (rabbit monoclonal IgG1 to IL-6; AffBiotech, Changzhou, China) in a humid chamber at room temperature overnight. The slides were washed again with TBST, incubated with a secondary peroxidase horseradish polymer-conjugated antibody (Histofine-HRP Nichirei, Tokyo, Japan) for 30 min at room temperature, washed with TBST, incubated with 3,3′ diaminobenzidine stain for 5 min, and counterstained with haematoxylin.

The Shapiro–Wilk test was used for testing data normality. Analysis of variance or Kruskal–Wallis tests, followed by Bonferroni or Dunn’s test were used for comparisons between groups. Differences were considered statistically significant at *p* < 0.05. Marginally significant results were further analysed using multiple independent *t*-tests. GraphPad v. 9.5.0 (GraphPad Software Inc. San Diego, CA, USA) and SPSS v.24.0.0 (Statistical Package for Social Sciences, IBM Corp., Armonk, NY, USA) were used for statistical analysis.

## 3. Results

**Figure 2:**
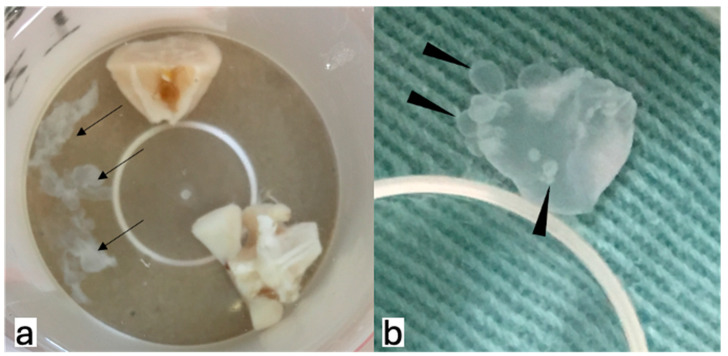
Cysts ((**a**), arrows) were identified in all three groups. Additionally, budding cysts were identified ((**b**), arrowheads) in one case in the Albendazole + Dexamethasone group.

**Figure 3:**
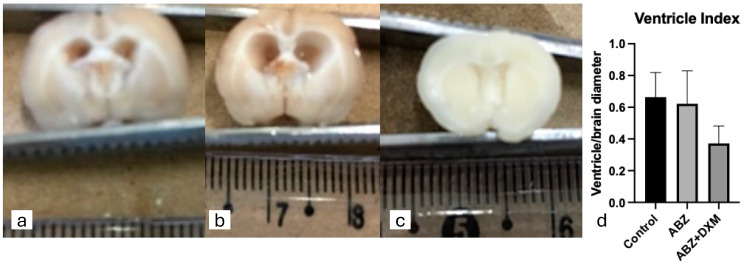
Examples of ventricle enlargements of animals from control (**a**), Albendazole (ABZ) (**b**), and Albendazole + Dexamethasone (ABZ + DXM) (**c**) groups. The median value was lower for the ABZ + DXM group, even though the difference did not reach statistical significance (**d**).

After collecting the brains, the cysts were still observed in all three groups and were distributed in the posterior fossa, basal cisterns, and inside the ventricles. Additionally, budding cysts were observed (Figure 2). The median ventricular indices for the control, ABZ, and ABZ + DXM groups were 0.68, 0.67, and 0.34, respectively (*p* = 0.08; Figure 3).

**Figure 4:**
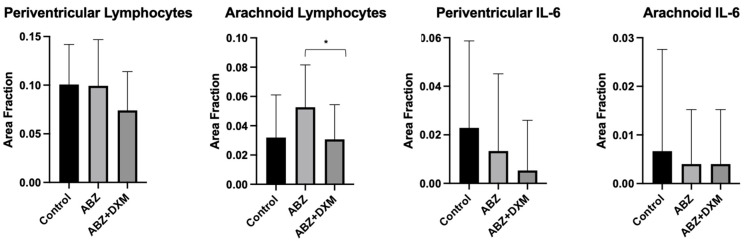
Comparison of the inflammatory features (lymphocytes and IL-6 immunoreactivity) on the periventricular and basal arachnoid regions. Albendazole + Dexamethasone demonstrated a tendency for less inflammation (bars as mean and standard deviation). *: *p* < 0.05.

**Figure 5:**
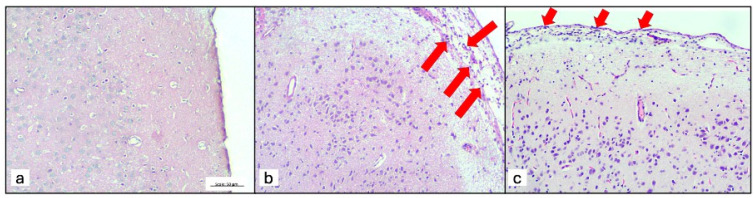
Inflammatory cells in the arachnoid region. The red arrows point to the presence of lymphocytes and ependymal disruption. (**a**) control; (**b**) Albendazole; and (**c**) Albenzadole + Dexamethasone groups.

A morphometric assessment revealed that the ABZ + DXM group, although not statistically significant, demonstrated a trend toward low inflammatory features (i.e., lymphocytes and immunoreactivity for IL-6) in the periventricular and basal arachnoid regions (Figure 4). A comparison between the ABZ and ABZ + DXM groups revealed a lower number of lymphocytes in the basal arachnoid region (Figure 5).

## 4. Discussion

Inflammation is the main pathogenic mechanism associated with EP-NCC and proper inflammation management is crucial for clinical management [15]. In P-NCC, as the parasites are surrounded by immunocompetent cells, the inflammatory response of the host against the parasite is quicker than that against EP-NCC. This may explain why patients with EP-NCC are older, as a longer incubation period exists between infection and symptom onset [16,17]. The CSF compartment is a privileged site for the parasite where it can remain hidden from the immunosurveillance of the host. EP-NCC presents with low lymphocyte reactivity accompanied by a regulatory response, which may be related to disease chronicity and low treatment responsiveness [18].

Treatment of EP-NCC usually requires several cysticidal cycles to successfully eliminate all parasites. Additionally, cysticidal treatment triggers symptoms related to inflammation, such as hydrocephalus, vasculitis, and cranial nerve injuries [15]. Therefore, anti-inflammatory drugs are strongly recommended [19], and corticosteroids are the most effective drugs for this purpose. However, long-term treatment causes frequent negative side effects related to corticosteroids, including diabetes, Cushing’s syndrome, and joint necrosis [20]. Additionally, their immunosuppressive effects may affect specific treatment efficacy. Innocuous and effective drugs to control inflammation are urgently required to treat EP-NCC [21]. Therefore, experimental models will be helpful for preclinical testing of novel therapeutic approaches, and the present model closely resembles that of human infection [13].

Our results were as expected: Albendazole treatment increased inflammatory features, probably due to damage to the cysticerci, which tends to be reduced with Dexamethasone [15,22,23]. Additionally, we demonstrated some new findings in this experimental model. First, we found a lower ventricle index in the ABZ + DXM group, implying reduced hydrocephalus. This finding aligns with the concept that hydrocephalus in the NCC is associated with mechanical obstruction and inflammation. Mechanical obstruction was still present as the parasites remained in the treatment groups, and ventricle size reduction was possibly related to reduced inflammation.

Second, viable parasites were detected in all the experimental groups. In this study, it was not possible to verify the efficacy of ABZ treatment, as the assessment was carried out two weeks after treatment. In humans, changes in the appearance of cysts on MRI may require several months for detection, and patients non-respondent to cysticidal drugs have been described [23]. A previous study on ABZ and DXM in treating intraperitoneal infection with *T. crassiceps* in mice demonstrated that concomitantly using DXM reduced the effectiveness of ABZ. However, this study used a higher ABZ dose (50 mg/kg), and the treatment was initiated 10 days after the infection [24]. Contrastingly, the treatment was initiated 3 months after the infection in our study. Future studies using the present model should analyse the morphological changes in the cyst membranes and the cyst protein content several weeks after terminating the treatment. Another relevant research group has found that the treatment of mice with intracranial inoculation of five cysts can induce morphological changes in the parasites within 24 h after the treatment [25,26]—even though it remains unclear whether long-term assessments could show an absence of parasites.

Finally, budding cysts were observed in the ABZ + DXM group. To the best of our knowledge, this is the first report of an intracranial budding cyst of *T. crassiceps* in *Rattus norvegicus.* This finding supports another similarity of the present experimental model to human EP-NCC in aberrantly proliferating cestode larvae (also known as racemose NCC) with new cysts sprouting [27]. This may be promoted by the parasite’s use of corticosteroid derivatives to synthesize hormones that enhance its reproductive capacity, as demonstrated by previous in vitro studies [28,29].

In conclusion, the treatment of the experimental model using *T. crassiceps* for subarachnoid inoculation is similar to that of human NCC, presenting the dual challenge of effectively destroying parasites in the CSF while controlling inflammation.

## Data Availability

The datasets generated during and/or analysed during the current study are available from the corresponding author upon request.
